# Mucosa-associated lymphoid tissue lymphoma of the trachea: case report

**DOI:** 10.1590/S1516-31802012000200010

**Published:** 2012-04-03

**Authors:** Maria Elisa Ruffolo Magliari, Renata Telles Rudge de Aquino, Anna Luiza Lobão Gonçalves, Fábio Marioni, Fabíola del Carlo Bernardi, Sérgio Brasil, Joaquim Antonio da Fonseca Almeida, Benedito Juarez Andrade, Carlos Sérgio Chiattone, Carlos Alberto da Conceição Lima

**Affiliations:** I MD, MSc. Instructor Professor, Discipline of Internal Medicine, Department of Clinical Medicine, Faculdade de Ciências Médicas da Santa Casa de São Paulo (FCMSCSP), São Paulo, Brazil.; II MD, PhD. Assistant Professor, Discipline of Internal Medicine, Department of Clinical Medicine, Faculdade de Ciências Médicas da Santa Casa de São Paulo (FCMSCSP), São Paulo, Brazil.; III Undergraduate Student, Faculdade de Ciências Médicas da Santa Casa de São Paulo (FCMSCSP), São Paulo, Brazil.; IV MD. First Attending Physician, Peroral Endoscopy Service, Irmandade da Santa Casa de Misericórdia de São Paulo, São Paulo, Brazil.; V MD, PhD. Assistant Professor, Discipline of Pathologic Anatomy, Department of Pathological Anatomy, Faculdade de Ciências Médicas da Santa Casa de São Paulo (FCMSCSP), São Paulo, Brazil.; VI MD, MSc. Second Attending Physician, Discipline of Hematology, Irmandade da Santa Casa de Misericórdia de São Paulo, São Paulo, Brazil.; VII MD. Instructor Professor, Discipline of Internal Medicine, Department of Clinical Medicine, Faculdade de Ciências Médicas da Santa Casa de São Paulo (FCMSCSP), São Paulo, Brazil.; VIII MD. First Attending Physician, Imaging Diagnostics Service, Irmandade da Santa Casa de São Paulo, São Paulo, Brazil.; IX MD, PhD. Adjunct Professor, Discipline of Hematology, Department of Clinical Medicine, Faculdade de Ciências Médicas da Santa Casa de São Paulo (FCMSCSP), São Paulo, Brazil.; X MD, PhD. Adjunct Professor, Discipline of Internal Medicine, Department of Clinical Medicine, Faculdade de Ciências Médicas da Santa Casa de São Paulo, São Paulo, Brazil.

**Keywords:** Lymphoma, B-Cell, marginal zone, Neoplasms, Tracheal neoplasms, Lymphoma, Lymphoma, non-Hodgkin, Linfoma de zona marginal tipo células B, Neoplasias, Neoplasias da traquéia, Linfoma, Linfoma não Hodgkin

## Abstract

**CONTEXT:**

Mucosa-associated lymphoid tissue (MALT) lymphomas are most commonly found in the stomach, lungs, orbital soft tissue, salivary glands and thyroid. Involvement of the trachea is extremely rare.

**CASE REPORT:**

This report describes a rare case of MALT lymphoma of the trachea in a 71-year-old woman who presented with a one-year history of coughing, dyspnea, hoarseness and weight loss. There was an infiltrative lesion in the mid-trachea. The anatomopathological diagnosis was only made from the fifth endoscopic biopsy attempt. Immunochemotherapy consisting of rituximab, cyclophosphamide, vincristine and prednisone (R-COP) induced complete remission of the symptoms and endoscopic lesion.

**CONCLUSIONS:**

MALT lymphoma of the trachea is extremely rare and indolent disease. It has to be considered in the differential diagnosis of airway lesions. It is crucial to obtain an anatomopathological diagnosis from a specialized pathologist. Immunochemotherapy with R-COP induced complete remission of the disease.

## RESUMO

**CONTEXTO:**

Linfomas de tecido linfoide associado à mucosa (TLAM) são mais comumente encontrados no estômago, pulmões, órbita, glandulas salivares e tireoide. O envolvimento da traqueia é extremamente raro.

**RELATO DE CASO:**

Relato de caso raro de linfoma TLAM de traqueia, em mulher de 71 anos com tosse, dispneia, rouquidão e emagrecimento há um ano. Detectou-se lesão infiltrativa na porção média da traquéia. O diagnóstico anatomopatológico só foi obtido na biópsia do quinto procedimento endoscópico. A imunoquimioterapia R-COP (rituximabe, ciclofosfamida, vincristina e prednisona) induziu remissão completa dos sintomas e da lesão endoscópica.

**CONCLUSÕES:**

Linfoma TLAM de traqueia é uma doença indolente e extremamente rara, que deve ser considerada no diagnóstico diferencial das lesões de vias aéreas. O diagnóstico anatomopatológico por um patologista experimentado é fundamental. Imunoquimioterapia com R-COP levou à remissão completa da doença.

## INTRODUCTION

Mucosa-associated lymphoid tissue (MALT) lymphomas are most commonly found in the stomach, lungs, orbital soft tissue, salivary glands and thyroid. Cases affecting the conjunctiva, breasts, kidney, skin, liver and prostate, and some involving bone marrow have been reported.^1^ Involvement of the trachea is extremely rare, with only fourteen cases found in the literature through a PubMed search with no time limit^2^^,^^3^^,^^4^^,^^5^^,^^6^^,^^7^^,^^8^^,^^9^^,^^10^^,^^11^^,^^12^^,^^13^^,^^14^^,^^15^ (Table 1).

This report describes a rare case of MALT lymphoma of the trachea in a 71-year-old woman who presented with a one-year history of coughing, dyspnea, hoarseness and weight loss. The diagnosis was made only on the fifth biopsy attempt, by means of bronchoscopy. Immunochemotherapy consisting of rituximab, cyclophosphamide, vincristine and prednisone (R-COP) induced complete remission of symptoms and the endoscopic lesion.

## CASE REPORT

A 71-year-old white woman presented with a one-year history of dry coughing, progressive dyspnea, 5 kg weight loss, nocturnal diaphoresis and six months of neck discomfort and hoarseness. She had a previous medical history of hypertension, which was under treatment with amlodipine (5 mg daily), and hypothyroidism treated with levothyroxine (50 mcg daily). She had had an episode of pneumonia, treated five years earlier. She had never smoked. She was anxious but looked well.

**Figure 1.**
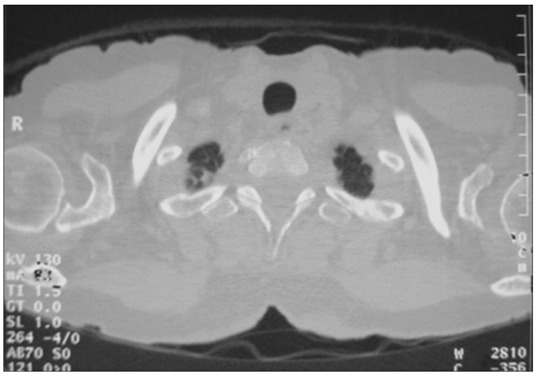
Tomography of the thorax showing irregularities and narrowing of the mid-trachea (arrow).

On physical examination, she presented reduced breathing sounds in both lungs, without any other remarkable finding. Renal and liver function tests, hemogram and lactic dehydrogenase levels were normal. Her beta 2 microglobulin level was 1.5 mg/l (upper limit of normal values: 1.31 mg/l). Chest x-ray showed a slight tracheal deviation, and lung function tests showed a mild obstructive picture. Bronchodilators and corticosteroids only attenuated the symptoms. Computed tomography (CT) of the chest (Figure 1) showed irregularities and narrowing of the mid-trachea. Bronchoscopy showed a friable infiltrative lesion involving all the walls of the trachea, starting 4 cm from the vocal cords and extending for 5 cm towards the carina, and stopping 2 cm away from it.

**Figure 2.**
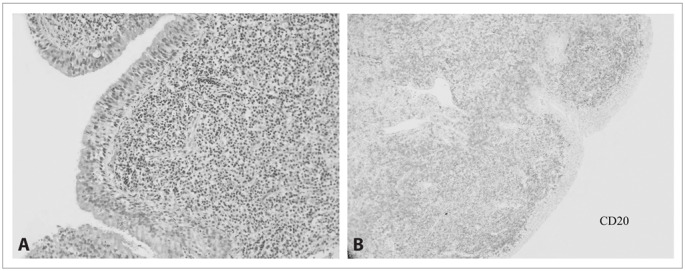
Photomicrograph of the tracheal wall. Panel A: showing heavy infiltration by lymphoma cells, consisting of small to medium sized lymphocytes with plasmacytic differentiation and lymphoepithelial lesions. Panel B: immunohistochemical staining for CD20 demonstrating diffuse infiltration of B cells in the chorion of the tracheal wall.

The initial biopsy showed reactional lymphoid hyperplasia. Only after the fifth endoscopic procedure with biopsies was the anatomopathological diagnosis made. The morphological and immunohistochemical features confirmed the presence of a low-grade non-Hodgkin’s B-cell lymphoma with plasmacytic differentiation, suggestive of MALT lymphoma. The immunohistochemical panel revealed that the lymphoma cells were positive for CD20, lambda and, rarely, CD138 and negative for CD43, cyclina and CD5 (Figure 2). A bone marrow biopsy was normal.

Treatment consisting of rituximab (375 mg/m^2^ once a week for four weeks) and prednisone (20 mg/day) was ineffective with no clinical response and worsening of the symptoms. R-COP immunochemotherapy was then started with the following doses: improved after vincristine was withdrawn in the second cycle. Bronchoscopy after the seventh cycle showed only a scar lesion, and this was confirmed in an anatomopathological sample. Eight cycles were completed with complete remission of symptoms.

**Table 1.** Results from our review of the medical databases. Date of search: April 12, 2011

Database	Search strategy	Results
PubMed	Lymphoma B cell marginal zone OR mucosa-associated lymphoid tissue OR MALT AND tracheal OR trachea	14 case reports^2-15^
Embase	Lymphoma B cell marginal zone OR mucosa-associated lymphoid tissue OR MALT AND tracheal OR trachea	5 case reports^3,9-11,15^

Using the same search strategies in the Cochrane Library, SciELO and Lilacs databases, no results were found.

## DISCUSSION

Primary tumors of the trachea are rare; squamous cell carcinoma and adenoid cystic carcinoma make up 75% of such cases.^2^^,^^3^ Hematopoietic tumors are rare and principally comprise plasmocytoma and non-Hodgkin’s lymphoma. The differential diagnoses for tracheal pathological conditions also include tuberculosis and fungal infections.

MALT consists of specialized lymphoid tissue protecting permeable mucosal sites that are in direct contact with the external environment. However, MALT lymphoma only occasionally occurs in places where this tissue is normally present, as seen in the tonsils and Peyer’s patches. It generally arises through acquisition as a result of some preexisting disorder. Examples are *Helicobacter pylori* colonization in the stomach, autoimmune disease in the salivary gland (Sjögren’s disease) and Hashimoto’s thyroiditis.^1^

The rarity of MALT lymphoma of the trachea is possibly due to the paucity of lymphoid tissue in this region. Kutta et al.^16^ studied 87 autopsies of the larynx in patients who were free from other diseases that might have affected laryngeal function. MALT was observed in the supraglottic region in 100% of the children, 90% or more of the adolescents, and 7.1% of individuals in their sixth decade. MALT was completely absent from the subglottis in all age groups. It is presumed that MALT occurs in the trachea in response to an antigenic stimulus, such as from bacterial or fungal infections and allergic reactions, but no antigens have been identified.^4^^,^^16^ MALT lymphomas are mainly located in the supraglottic and glottic areas, with only one reported case in the subglottic region.^9^

The clinical picture is similar to that of chronic obstructive pulmonary disease, which often delays the diagnosis. Our patient received treatment for asthma with minimal improvement in symptoms, probably due to steroids. Bronchoscopy and biopsy are essential. Most cases present with a polypoid lesion. In our case, there was some difficulty in obtaining a sample that would be satisfactory for the anatomopathological analysis. Only in the fifthbronchoscopy was enough material obtained. The same problem has been described in another problematic case,^3^ and such occurrences may represent a characteristic of the disease. The possibility that treatment can be implemented reinforces the need to obtain an anatomopathological diagnosis.

At presentation, MALT lymphoma is generally localized. The course tends to be indolent and the prognosis is good.^2^^,^^4^ In a series of 75 cases of non-gastrointestinal MALT lymphoma, there was complete remission in 79% and partial remission in 21%. The best results were found for thyroid and tear duct tumors, while skin tumors were least responsive.^1^

There are no clear guidelines for the treatment of MALT lymphoma, with different therapeutic options depending on the site.^13^ When located in the trachea, a diversity of treatments have been shown to be effective: surgical resection, radiotherapy, bronchoscopic therapy, chemotherapy, immunotherapy (rituximab) and immunochemotherapy.^1^^,^^2^^,^^3^^,^^4^^,^^5^^,^^6^^,^^7^^,^^8^^,^^9^^,^^10^^,^^11^^,^^12^^,^^13^^,^^14^^,^^15^ The treatment can be done in combination or separately. Antibiotic therapy is not indicated, since no antigens like *Helicobacter pylori* are implicated in the pathophysiology.

## CONCLUSION

MALT lymphoma of the trachea is an extremely rare and indolent disease. It has to be considered in the differential diagnosis of airway lesions. It is crucial to obtain an anatomopathological diagnosis from a specialized pathologist.
